# Porphyrin-magnetite nanoconjugates for biological imaging

**DOI:** 10.1186/1477-3155-9-13

**Published:** 2011-04-08

**Authors:** Małgorzata Nowostawska, Serena A Corr, Stephen J Byrne, Jennifer Conroy, Yuri Volkov, Yurii K Gun'ko

**Affiliations:** 1 School of Chemistry, Trinity College Dublin, College Green, Dublin 2, Ireland; 2 Department of Clinical Medicine, Trinity Centre for Health Sciences, Trinity College Dublin, St. James's Hospital, Dublin 8, Ireland; 3 School of Physical Sciences, University of Kent, Canterbury, CT2 7NH

## Abstract

**Background:**

The use of silica coated magnetic nanoparticles as contrast agents has resulted in the production of highly stable, non-toxic solutions that can be manipulated *via *an external magnetic field. As a result, the interaction of these nanocomposites with cells is of vital importance in understanding their behaviour and biocompatibility. Here we report the preparation, characterisation and potential application of new "two-in-one" magnetic fluorescent nanocomposites composed of silica-coated magnetite nanoparticles covalently linked to a porphyrin moiety.

**Method:**

The experiments were performed by administering porphyrin functionalised silica-coated magnetite nanoparticles to THP-1 cells, a human acute monocytic leukaemia cell line. Cells were cultured in RPMI 1640 medium with 25 mM HEPES supplemented with heat-inactivated foetal bovine serum (FBS).

**Results:**

We have synthesised, characterised and analysed *in vitro*, a new multimodal (magnetic and fluorescent) porphyrin magnetic nanoparticle composite (PMNC). Initial co-incubation experiments performed with THP-1 macrophage cells were promising; however the PMNC photobleached under confocal microscopy study. β-mercaptoethanol (β-ME) was employed to counteract this problem and resulted not only in enhanced fluorescence emission, but also allowed for elongated imaging and increased exposure times of the PMNC in a cellular environment.

**Conclusion:**

Our experiments have demonstrated that β-ME visibly enhances the emission intensity. No deleterious effects to the cells were witnessed upon co-incubation with β-ME alone and no increases in background fluorescence were recorded. These results should present an interest for further development of *in vitro *biological imaging techniques.

## Background

Magnetic nanoparticles have been the focus of much research due to their potential biomedical applications as both diagnostic tools and therapeutic agents [1,2]. Suspensions of superparamagnetic nanoparticles of iron oxide are promising magnetic resonance imaging contrast agents, improving the image quality of anatomical structures by altering the relaxation time of the protons present [3-6]. Magnetic nanoparticles may also induce heat once subjected to an external magnetic AC field, opening up the possibility of hyperthermic cancer treatment [7,8]. Site-specific drug delivery is an enticing possibility, which may be realised by loading nano-magnetic carriers with therapeutic agents and directing them to the site of interest using external magnetic fields [9,10]. The assembly of a number of building blocks with different functionalities could provide a multimodal platform allowing for the combination of diagnostic imaging and therapeutic capabilities [11-13]. In particular, nanoscale entities combining magnetic and fluorescent properties have attracted much attention. Their potential uses in medicine are far-reaching including in imaging, bio- and chemo- sensing, drug delivery and therapy systems. Challenges remain in their fabrication, which frequently involve multi-step reactions to prevent the quenching of the fluorophore. Several synthetic routes have been reported, including core-shell composites, bilipid layers between the particle surface and the fluorescent moiety composites, and use of electrostatic interactions between stabilizers, magnetic particles and fluorophores [14-18]. The surface chemistry of these composite materials plays a crucial role in cellular uptake. For example magnetic nanoparticles, functionalised with a chitosan-labelled fluorescein isothiocyanate derivative, have shown uptake by human hepatoma cells *via *charged interactions [19]. Interesting multifunctional nanocomposites comprising of silver and iron oxide nanoparticles embedded in a silica shell, together with a Raman reporter molecule have been published by Murphy *et. al*. [20]. The introduction of a rhodamine moiety to the silica surface gives the composite a broad range of potential applications due to its magnetic, light scattering, SERS and fluorescent properties. Trapping a rhodamine dye within a silica matrix during the formation of a shell surrounding the magnetite nanoparticles has also led to the formation of magnetic-fluorescent nanocomposites [21,22]. In addition superparamagnetic iron oxide nanoparticles (SPION) are of particular interest for targeted cancer therapy. Tumour-targeted hyperthermia using super-paramagnetic, biocompatible, and nanosized delivery vehicles would allow patients to receive increased treatment dosages while minimizing side effects. Some recent research investigating SPION was also dedicated to the early detection of cancer, diabetes, and atherosclerosis [23-25].

Here we report the preparation, characterisation and application of new "two-in-one" magnetic-fluorescent nanocomposites composed of silica-coated magnetite nanoparticles, which are covalently linked to a porphyrin moiety.

## Results and discussion

### Synthesis and characterisation of the porphyrin-magnetite nanocomposite (PMNC)

**Figure 1:**
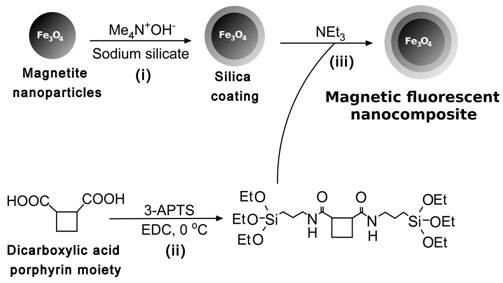
**PMNC synthesis: Preparation of the PMNC using a base catalyzed condensation reaction to attach a 3-APTS modified protoporphyrin IX to silica coated magnetite nanoparticles**. (i) A thin silica layer is introduced on the magnetite nanoparticle surface by employing TMAH and sodium silicate. (ii) An amide coupling reaction is performed between the carboxylic acid groups of protoporphyrin IX and 3-APTS. (iii) A base catalysed hydrolysis between the modified porphyrin and the silica coated magnetite nanoparticles results in a magnetic-fluorescent nanocomposite.

Magnetite nanoparticles have been produced by a previously reported co-precipitation method (see Materials and Methods) [26,27]. Application of a silica layer was achieved by following a method reported by Philips and co-workers [28]. Briefly, a colloidal solution of magnetite nanoparticles in tetramethylammonium hydroxide (TMAH) was treated with sodium silicate in order to deposit a thin layer of silica on the surface of the oxide particles (Figure 1). In a separate step, a carboxylic acid protoporphyrin (protoporphyrin IX) was reacted with 3-aminopropyltriethoxysilane (3-APTES) under inert conditions in the presence of the carbodiimide coupling agent (EDCI) to form an amide bond. This modified porphyrin was then reacted with the silica coated particles, to form a stable colloidal suspension. In this case the silica shell was necessary to provide an effective barrier between the particle core and the fluorescent porphyrin, preventing the risk of quenching.

Elemental analysis of the PMNC shows a content of C - 9.31, H - 1.21 and N - 1.51%. This clearly confirms the presence of conjugated porphyrin molecules at the surface of nanoparticles.

**Figure 2:**
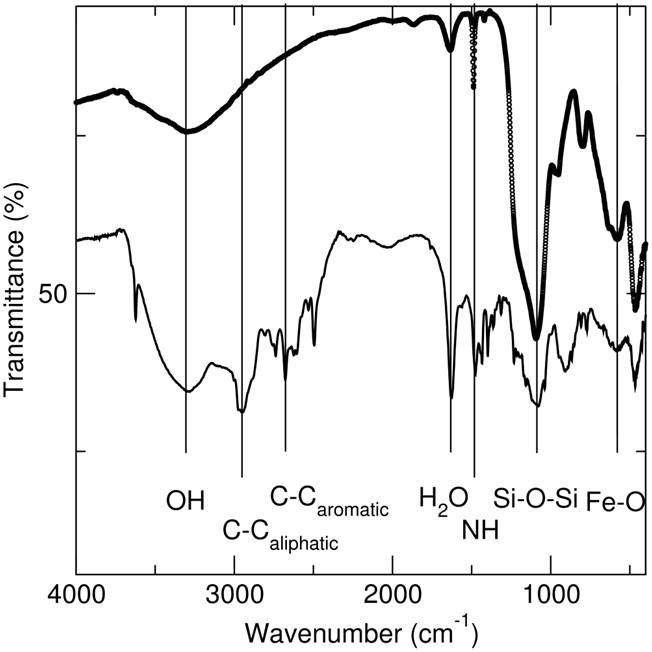
**FTIR spectra of the silica coated magnetite nanoparticles before and after reaction with the protoporphyrin were recorded in KBr**. The spectrum of silica coated particles is shown in heavy black circles above.

The presence of the porphyrin on the surface of the silica-coated magnetite nanoparticles was also confirmed by Fourier Transform Infrared Infra-red (FTIR) spectroscopy (Figure 2). FTIR spectra of the silica coated magnetite nanoparticles before and after reaction with the protoporphyrin were recorded in KBr. The silica coated particles (shown in heavy black circles above) have stretches at 579, 1087, 1625 and 3286 cm^-1 ^corresponding to the Fe-O, Si-O-Si, and water stretches respectively. The stretch found at 947 cm^-1 ^is due to the presence of Fe-O-Si bonds in the sample. For the nanocomposite material, again Fe-O and water stretches are found at 579, 1621 and 3286 cm^-1 ^respectively. The Fe-O-Si stretch is this time masked by the NH wagging from the porphyrin centred at 901 cm^-1^. NH stretches are found at 1473 cm^-1^. Aromatic and aliphatic stretches associated with the porphyrin are found at 2500 and 2945 cm^-1 ^respectively. FTIR spectra (Figure 2) of the porphyrin nanocomposite material showed stretches for NH due to the presence of the porphyrin at 901 and 1473 cm^-1^. Aromatic and aliphatic stretches associated with the porphyrin are found at 2500 and 2945 cm^-1 ^respectively. A core-shell-like morphology is apparent from TEM images taken of the silica coated nanoparticles. The average primary particle size is calculated to be 9.3 ± 1 nm. The silica coating on the particles has an average thickness of 3 nm, however upon reaction with the 3-APTES modified protoporphyrin IX, the coating thickness is found to slightly increase to 4 nm.

**Figure 3:**
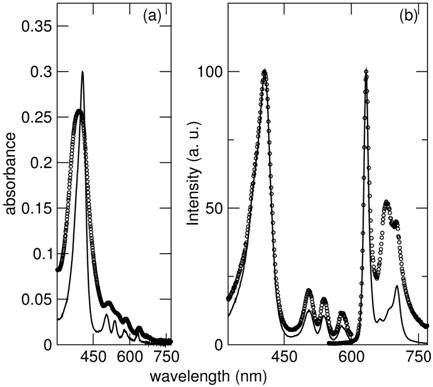
**Optical characterisation of the PMNC**: (a) Absorbance spectra of original protoporphyrin IX (solid line) [1.57 × 10^-6 ^M] and PMNC (dotted line) [100 μl PMNC in 3 ml THF]. (b) Normalised emission and excitation spectra of original porphyrin (solid line) [5.23 × 10^-7 ^M] and PMNC (dotted line) [100 μl PMNC in 3 ml with THF]. λ_em _= 633 nm, λ_ex _= 406 nm.

Absorbance spectra of the original porphyrin and the PMNC were taken in tetrahydrofuran, THF (Figure 3). Peaks at 406, 505, 538, 577 and 632 nm were observed for a solution of porphyrin. Emission (λ_ex_, 406 nm) and excitation spectra (λ_em _was 633 nm) have also been recorded, with significant broadening witnessed for the PMNC when compared to the original porphyrin. This can be attributed to the scattering of the incoming light by the PMNC suspension, but gives further indication of the binding of the porphyrin to the nanoparticle surface.

**Figure 4:**
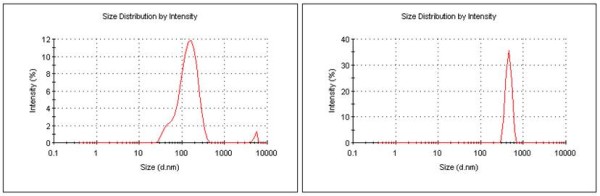
**DLS results for suspensions of (a) silica coated magnetite nanoparticles and (b) silica-coated porphyrin functionalised magnetite nanoparticles**.

Dynamic Light Scattering (DLS) studies of silica coated magnetite nanoparticles have shown a very broad size distribution with an average hydrodynamic diameter of 131 nm, due to aggregation of nanoparticles. However, silica coated porphyrin functionalised magnetic nanoparticles have demonstrated relatively narrow distribution with an average hydrodynamic diameter of 818 nm (Figure 4).

**Figure 5:**
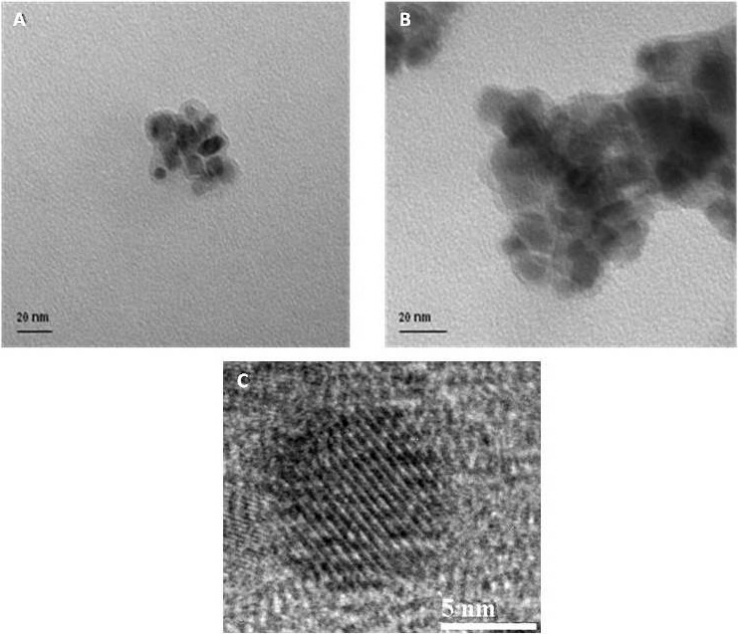
**TEM images (a) silane coated nanoparticles and (b) after reaction with protoporphyrin and (c) HR TEM image of the porphyrin coated magnetite nanoparticle**.

TEM images taken of the silica coated nanoparticles reveal the presence of a core-shell structure (Figure 5). The silane coating on the particles has an average thickness of 3 nm and the particles are found to aggregate in group of approximately ten particles. Upon reaction with the 3-aminopropyltriethoxysilane modified protoporphyrin, the coating thickness is found to slightly increase to 4 nm.

**Figure 6:**
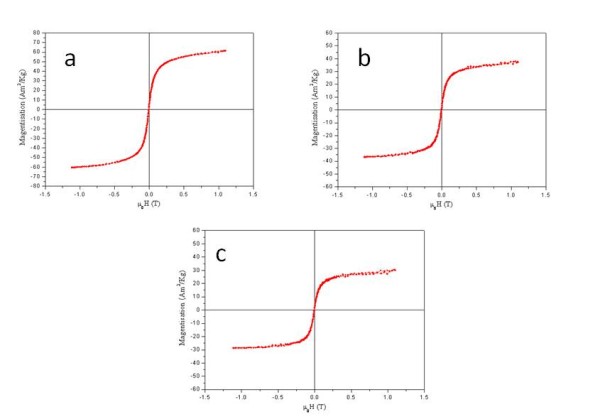
**Magnetisation curves of original non-coated (a) magnetitite nanoparticles, (b) silica coated magnetite nanoparticles and (c) silica-coated porphyrin functionalised magnetite nanoparticles**.

Magnetisation measurements of original non-coated magnetite nanoparticles, silica coated magnetite nanoparticles and silica-coated porphyrin functionalised magnetite nanoparticles as expected, have shown a gradual reduction in saturation magnetisation values (58.4 Am^2^/kg for pristine magnetite nanoparticles, 35.2 Am^2^/kg for silica coated magnetite nanoparticles and 27.5 Am^2^/kg for the corresponding porphyrin conjugates) with an increasing amount of non-magnetic material, i.e. silica and porphyrin conjugates (Figure 6).

The combination of both magnetic and fluorescent properties in one nanocomposite can provide additional benefits. For example, these nanocomposites could serve as multimodal assays for *in vitro*- and *vivo*- bioimaging applications such as MRI and fluorescence microscopy. They can also be used as bimodal agents for anticancer therapy, encompassing photodynamic and hyperthermic capabilities. Fluorescent-magnetic nanocomposites may also be utilised as a multimodal diagnostic and therapeutic tool, which could be used, for example, to identify, diagnose and simultaneously treat various diseases. Other potential exciting applications of these nanocomposites include cell tracking, cytometry and magnetic separation, which could be easily controlled and monitored using fluorescent microscopy and MRI.

### Cell culture experiments

Previously, the cytotoxic effects of poly(ethylene glycol)-co-fumarate (PEGF)-coated magnetite nanoparticles and their capability for changing cell culture medium compositions have been investigated. The primary mouse connective tissue cells (L929) were used for this purpose. Toxicity levels and changes in the cell culture medium compositions were determined using the MTT assay and a UV/vis spectroscopy method, respectively. As compared to the conventional method, a more reliable method for determining the cytotoxicity of nanoparticles for *in vitro *applications is found [29]. It also has been demonstrated that the incorporation of anticancer drugs such paclitaxel into PLGA nanoparticles in nanoparticles strongly enhances the cytotoxic effect of the drug as compared to Taxol, this effect being more relevant for prolonged incubation times [30,31].

**Figure 7:**
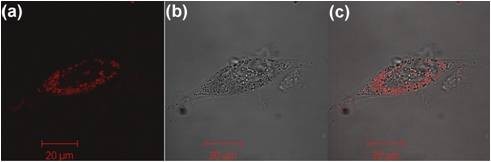
**THP-1 cells incubated with the PMNC: THP-1 cells incubated with the PMNC for 3 hours**. (a) confocal (b) bright field and (c) composite images of a THP-1 cell with internalised PMNC (λ_ex _= 540 nm).

In our studies THP-1 (human acute monocytic leukaemia) cells were cultured in the presence of phorbol 12-myristate 13-acetate (PMA) for 3 days to initiate monocyte to macrophage differentiation. Half the volume of complete culture media was then replaced with serum free media to "starve" the cells, allowing for an enhanced up-take of the PMNC. Figure 7 shows a confocal image of a THP-1 cell after incubation with the PMNC for 3 hours. The particles are localised in the cytoplasm, as detected by confocal imaging, and the image is representative of the broader cell population.

**Figure 8:**
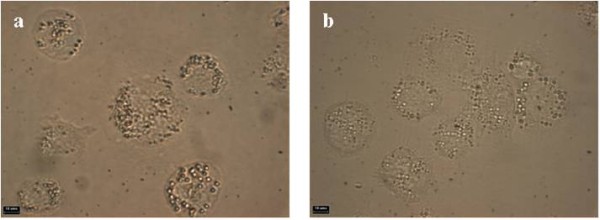
**Transmitted light microscopic images of (a) untreated THP-1 cells and (b) THP-1 cells in the presence of β-ME**. Note scale bar equals 10 μm. No deleterious effects to the cell size, shape or membrane integrity are noted.

During the experiments we have found that following an exposure time of only 482 ms, the PMNC begins to photobleach, and after only 4 seconds, the fluorescence intensity has greatly decreased (Figure 8). This process is highly undesirable as it makes the generation, capture and interpretation of the image extremely difficult and does not allow for a full examination of the cells to determine precisely the nature of the nanocomposite localisation.

**Figure 9:**
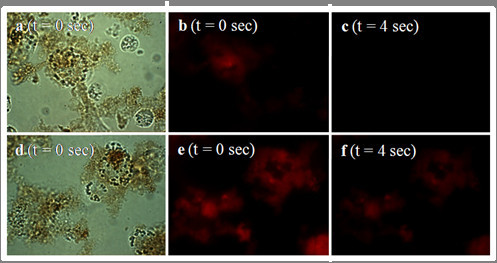
**Evaluation of PMNC emission intensities: comparison of the PMNC emission intensity when incubated with THP-1 cells with (bottom panel) and without (top panel) β-ME**. (a) Bright field and (b, c) fluorescence images of the PMNC and the THP-1 cells without β-ME (d) Bright field and (e, f) fluorescence images of the PMNC and the THP-1 cells with β-ME. **Note: **(a, b, d, e) images taken at t = 0 seconds, (c, f) taken at t = 4 seconds, exposure time for each image is 482 ms and (λ_ex _= 540 nm).

**Figure 10:**
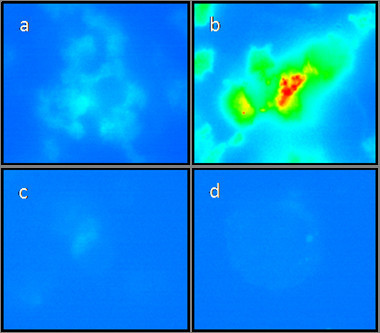
**Pseudocoloured fluorescence images: pseudocolored images of (a) THP-1 cells incubated with the PMNC (b) THP-1 cells incubated with PMNC in the presence of β-ME (c) untreated THP-1 cells and (d) cells in the presence of β-ME alone**. Transition colour from blue to red indicates an increase in fluorescence intensity.

In an effort to alleviate the photobleaching, β-mercaptoethanol (β-ME) was employed to counteract this problem, and was added once the cells were fixed. Control experiments performed with THP-1 cells in the presence of β-ME show that no deleterious effects are witnessed and the cells retain their size, shape and membrane integrity (Figure 9). Addition of β-ME to cells cultured with the PMNC prevented this photobleaching effect and resulted in a stronger fluorescence signal compared to those without β-ME (see Figures 9 and 10). Following the same cell preparation and co-incubation times, we can clearly see not only an increased resistance to photobleaching, but the addition of β-ME also resulted in an enhanced and extended fluorescence emission. This improved photostability is due to the β-ME acting as a scavenger for singlet oxygen, which is traditionally attributed to the photobleaching of porphyrin based dyes.

To further exemplify the enhancement in fluorescent emission intensity due to the presence of the β-ME, pseudocoloured images of the cells were generated (Figure 10). The pseudocoloured images reflect the integrated fluorescence intensity levels across the cytoplasmic and nuclear compartments of the THP-1 cells. Background fluorescence appears blue and predominates for those cells without any nanocomposite added (panels 10 c and d) but also for cells cultured with the PMNC alone (panel 10 a). A dramatic change is witnessed upon addition of the β-ME (panel 10 b). The PMNC is no longer quenched so dramatically and readily emits with substantial emission increases recorded after 4 seconds.

To verify that the increased emission is due to the PMNC and not as a result of increased cellular autofluorescence, control experiments were also conducted. Panels 10 c and d show untreated and β-ME treated THP-1 cells respectively and clearly illustrate that the level of background fluorescence remains constant throughout the experiment. This proves that autofluorescence does not contribute to the enhanced levels of emission witnessed for those cells cultured with the PMNC in the presence of β-ME.

**Figure 11:**
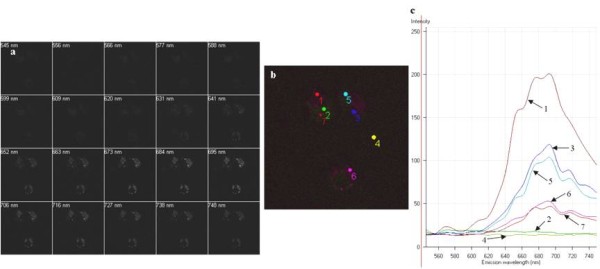
**Lambda scan of THP-1 cells and PMNC taken using a Zeiss LSM Meta-510 confocal microscope**. (a) Series of images recorded for various wavelength ranges between 545-748 nm. (b) Merge of all images from (a) showing the slight variations in λ across the sample. (c) Shows the spectra for each of the region of interest (ROI) in b. Excitation wavelength 405 nm.

**Figure 12:**
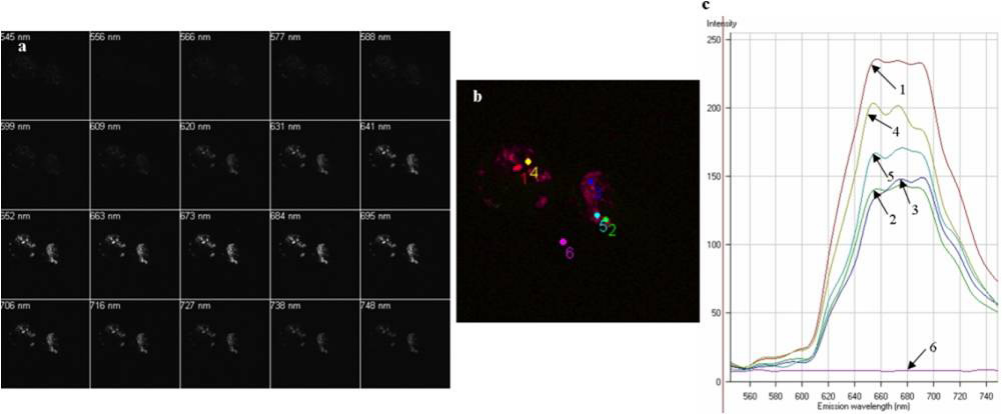
**Lambda scan of THP-1 cells and PMNC in the presence of β-ME taken using Zeiss LSM Meta-510 confocal microscope**. (a) Series of images recorded for various wavelength ranges between 545-748 nm. (b) Merging of all images from (a) showing the variations in λ across the sample. (c) Emission spectra for each point mapped in (b).

In order to probe the maximum fluorescence intensity of the PMNC as a function of excitation wavelength and also to monitor the fluorescence intensity across the cell compartments, a series of fluorescence images were recorded over varying wavelengths together with emission spectral mapping of the cell. Cells were cultured with the PMNC with and without the presence of β-ME. Lambda scans recorded of cells cultured without β-ME show a noted decrease in brightness and the combined image is noticeably lower in fluorescence intensity. This is shown quantitatively in the fluorescence emission spectra, which show an almost 20% decrease in fluorescence intensity (Figures 11 and 12).

**Figure 13:**
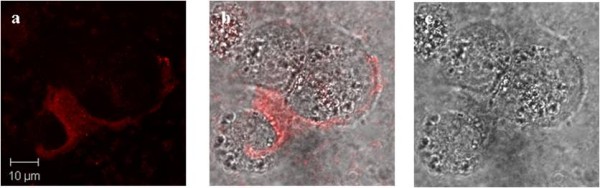
**(a) Confocal, (b) composite and (c) bright field images showing the localization patterns of the PMNC in live THP-1 macrophage cells following a 24 hour co-incubation period**. Panel (a) shows the clear fluorescence labelling of the outer cell membranes by the nanoparticles. The PMNC did not penetrate the cell membrane as these cells were not incubated with serum free media.

**Figure 14:**
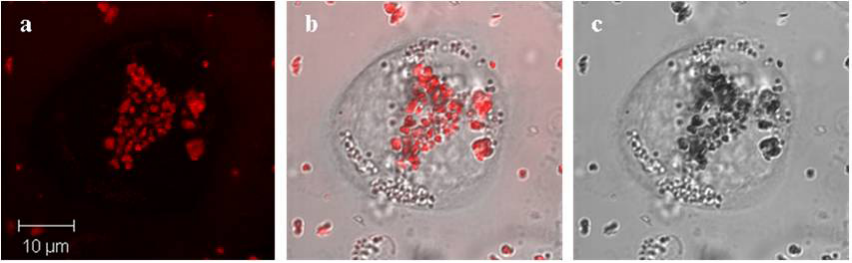
**(a) Confocal, (b) composite and (c) bright field images of THP-1 macrophage exposed to the PMNC for 24 hours**. Cells were incubated with serum free media for 2 hours.

**Figure 15:**
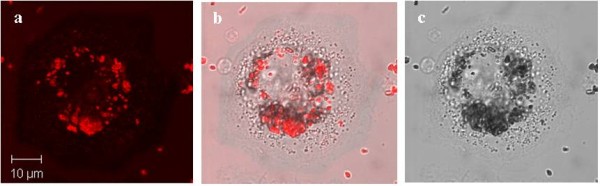
**(a) Confocal, (b) composite and (c) bright field images of THP-1 macrophage cells exposed to the PMNC for 48 hours**. Cells were incubated with serum free media for 2 hours.

**Figure 16:**
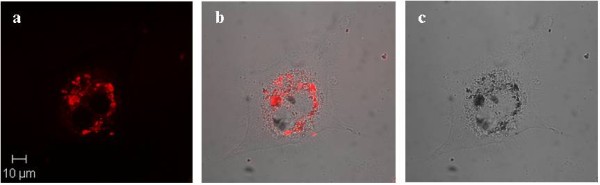
**(a) Confocal, (b) composite and (c) bright field images of THP-1 macrophage cells exposed to the PMNC for 5 days**. Cells were incubated with serum free media for 2 hours.

Finally, to compare the results a series of similar control experiments have been performed on "non-starved" THP-1 cells, which were incubated with serum free media. Confocal and composite images of the cells incubated with fluorescent magnetic nanoparticles have been taken at different periods of time 24 hrs, 48 hrs and 5 days (Figures 13, 14, 15 and 16). The results have demonstrated that there was no cellular uptake of the fluorescent nanocomposites but only binding of the PMNC to the outer cell membranes.

## Conclusion

In summary, we have successfully synthesised a new two-in-one magnetic-fluorescent nanocomposite. Initial co-incubation experiments performed with THP-1 macrophage cells in the presence of the PMNC showed a distinct photobleaching of the porphyrin upon exposure to light under a fluorescent microscope. This bleaching was counteracted by the addition of β-ME which resulted not only in increased fluorescence intensity levels, but also allowed for enhanced imaging and increased exposure times of the PMNC in a cellular environment. This was further corroborated by pseudofluorescent images which show how β-ME visibly enhances the emission intensity. Furthermore, no deleterious effects to the cells were witnessed upon co-incubation with β-ME alone and no increases in background fluorescence were recorded. We believe that this research should present an interest for further development of and new multimodal nanoparticulate agents for *in vitro *biological imaging.

## Materials and methods

### Synthesis

#### Synthesis of nanoparticles and coating with silica

Magnetite nanoparticles were prepared according to the previously reported coprecipitation method. Stock solutions of 25 mL of each of 1 M FeCl_3_.6H_2_O (6.56 g, 0.024 mol), 0.5 M FeCl_2_.4H_2_O (2.48 g, 0.012 mol), and 0.4 M HCl were made up with Millipore water. NaOH solution (0.5 M, 250 mL) was heated to 80°C and the iron solution was added dropwise. After one hour of stirring (at 1200 rpm) with heat, the black magnetic nanoparticles were washed with Millipore water (5 × 20 mL) until neutral. A sample of the nanoparticles (1 g) was dispersed in tetramethylammonium hydroxide (30 mL) and made up to 570 mL with Millipore water. 0.58% activated sodium silicate (430 mL) was added to the nanoparticle suspension. The sodium silicate was activated by passing through a regenerated cation exchange resin. After two hours stirring, the magnetic fluid was transferred into dialysis tubing and dialysed against Millipore water brought to pH 10 by the addition of tetramethylammonium hydroxide. The dialysis tubing was prepared by boiling tubing of the required length in 2% (w/v) sodium bicarbonate (100 mL) and 1 mM EDTA (100 mL) at pH 8.0 for ten minutes. The tubing was rinsed with Millipore water then boiled for ten minutes in 1 mM EDTA followed by Millipore water rinsing. After 24 hour dialysis, the solution was brought to pH 8.0 by the addition of dilute HCl. The particles were washed and finally dried under vacuum.

#### Modification of protoporphyrin and attachment to magnetic nanoparticles

Protoporphyrin IX (0.1 g; 0.177 mmol) was dissolved in dry THF (20 mL). *N*-(3-Dimethylaminopropyl)-*N*'-ethylcarbodiimide (0.17 g; 0.887 mmol) was added to' the stirring deep red solution and the reaction mixture was stirred (at 1200 rpm) for 3 hours at 0°C. The reaction was carried out in the dark to prevent any possible photobleaching of the porphryin. 3-Aminopropyltriethoxysilane (83 μL; 0.355 mmol) in 2 mL dry THF was added to the porphyrin solution at 0°C. The reaction mixture was brought to room temperature and allowed to react overnight. Silica coated magnetite nanoparticles (0.1 g) were added to the reaction vessel with triethylamine (300 μL) and allowed to react for 24 hours. The mixture was centrifuged and the particles were washed with bench THF (5 × 40 mL) to remove any un-reacted porphyrin or unwanted side-products. The particles of PMNC were finally washed with water. Elemental analysis, found: C - 9.31, H - 1.21, N - 1.51.

### Tissue culture

THP-1 cells, a human acute monocytic leukaemia cell line, were obtained from the American Type Culture Collection (ATCC, LGC Promochem, U.K.). Cells were cultured in RPMI 1640 medium with 25 mM HEPES (Gibco, Invitrogen Ltd., Ireland), supplemented with heat-inactivated foetal bovine serum (FBS) (9%), 200 mM/ml L-glutamine (0.9%, 1.8 mM/ml), 10,000 U/ml penicillin (0.9%, 90 U/ml), and 10 mg/ml streptomycin (0.9%, 0.09 mg/ml) at 37°C and 5% CO_2 _content.

### Cell assays

THP-1 cells were seeded into an 8-well chamber slide, with a final concentration of 2 × 10^4 ^cells per well. To induce monocyte to macrophage differentiation, THP-1 cells were cultured in 100 ng/ml phorbol 12-myristate 13-acetate (PMA) for 3 days at 37°C in 5% CO_2_. Half the volume of media was then replaced by serum free media (RPMI 1640 with 25 mM HEPES) and the cells were incubated for 2 hours at 37°C in 5% CO_2_. The PMNC was then added at a final concentration of 0.25 mg/ml. To another well, β-mercaptoethanol β-ME was added. A control experiment with the Protoporphyrin IX dissolved in mixture of water and tetrahydrofuran (THF) was carried out in parallel using the same conditions, with a final porphyrin concentration of 0.119 mg/ml. Fluorescence images were recorded using a Nikon TE3000 inverted microscope. Confocal imges were collected using a 63× oil immersion objective, laser excitation 561 nm and LP 575 nm filter on a Zeiss LSM Meta-510 confocal microscope.

### Spectroscopy

UV-Vis absorption spectra were recorded using a Cary 50 UV-Vis spectrophotometer. Fluorescence measurements were performed on a Cary Eclipse spectrophotometer. FT-IR spectra were recorded KBr using a Perkin Elmer Spectrum One FT-IR spectrophotometer. TEM images were taken on a Hitachi H-7000 at a beam voltage of 100 kV.

## Abbreviations

AC: alternating current; 3-APTES: 3-aminopropyltriethoxysilane; β-ME: β-mercaptoethanol; DLS: Dynamic Light Scattering; EDTA: ethylenediamine tetraacetic acid; EDCI: 1-ethyl-3-(3-dimethylaminopropyl carbodiimide); HEPES: 4-(2-hydroxyethyl)-1-piperazineethanesulfonic acid; THP-1 cells: human acute monocytic leukaemia cells; PMA: phorbol 12-myristate 13-acetate; PMNC: porphyrin-magnetite nanocomposite; RPMI: Roswell Park Memorial Institute Medium; TEM: Transmission electron microscopy; THF: tetrahydrofuran; TMAH: tetramethylammonium hydroxide; UV-vis: Ultra-violet visible.

## Competing interests

The authors declare that they have no competing interests.

## Authors' contributions

MN and JC performed all cellular experiments. MN and SAC performed all nanoparticle synthesis and characterisation, and wrote the manuscript with SB and YKG. MN and JC conducted confocal imaging. YKG and YV designed the overall project and helped with data and manuscript revision. All authors read and approved the final manuscript.
